# Gut microbiota of *Suncus murinus*, a naturally obesity-resistant animal, improves the ecological diversity of the gut microbiota in high-fat-diet-induced obese mice

**DOI:** 10.1371/journal.pone.0293213

**Published:** 2023-11-22

**Authors:** Mingshou Zhang, Ting Yang, Rujia Li, Ke Ren, Jun Li, Maozhang He, Juefei Chen, Shuang-Qin Yi

**Affiliations:** 1 Department of Frontier Health Sciences, Graduate School of Human Health Sciences, Tokyo Metropolitan University, Tokyo, Japan; 2 Faculty of Physical Education, Qujing Normal University, Qujing, Yun Nan, China; 3 State Key Laboratory of Oncogenes and Related Genes, Shanghai Cancer Institute, Ren Ji Hospital, School of Medicine, Shanghai Jiao Tong University, Shanghai, China; 4 Department of Microbiology, School of Basic Medical Sciences, Anhui Medical University, Hefei, China; UAE University: United Arab Emirates University, UNITED ARAB EMIRATES

## Abstract

**Background:**

The global population of obese individuals is increasing, affecting human health. High-fat diets are a leading cause of this epidemic, and animal models, such as mice, are often used in related research. Obese individuals have a different gut microbiota composition from non-obese ones, characterized by a sizeable population of certain bacteria associated with fat storage. The gut microbiome plays a significant role in regulating human physiological and metabolic functions. Links between obesity, high-fat diets and gut microbiota have become hot topics of discussion. Recently, research on the modulation of the gut microbiota has focused on fecal microbiota transplantation (FMT), which has been recognized as an effective method of studying the function of gut microbiota.

**Objectives:**

The purpose of this study was to investigate how the gut microbiota of *Suncus murinus*, a naturally obesity-resistant animal, through FMT, affected the ecology of the gut microbiota of high-fat diet induced obese mice.

**Methods:**

In this study, *Suncus murinus* was used as a donor for FMT. High-fat diet induced C57BL/6NCrSIc mice were used as recipients, the body weight changes were measured and changes in their gut flora were analyzed using a 16S rRNA gene analysis.

**Results:**

The study found that, after the FMT procedure, the FMT group tended to have a lower body weight than the control group. At the phylum level, the most predominant phyla in all groups were Firmicutes and Proteobacteria, while Deferribacteres was not detected in the FMT or antibiotic administration groups, and Bacteroidetes was not present in the antibiotic administration group. At the genus level, the FMT group had significantly lower OTU richness than the control group but greater diversity than the control group.

**Conclusions:**

These results indicate that FMT from *Suncus murinus* can help reorganize and improve the gut microbiota of mice in a balanced and diverse ecosystem.

## Introduction

The prevalence of obesity has been on the rise globally, leading to significant negative impacts on physical health in recent years. Various theories exist to explain the causes of obesity, and one notable factor is the shift in people’s dietary patterns, with high-fat diets (HFD) being a particular concern [1]. Currently, some animal models, especially mouse models, are widely used in research on HFD-induced obesity [2, 3]. Studies have shown that obese individuals tend to have a different composition of gut microbiota from those who are lean, with a lower diversity of gut bacteria and an overrepresentation of certain types of bacteria that are associated with increased calorie extraction and storage of fat [4]. Links between obesity, HFD and gut microbiota have thus become hot topics of discussion.

The microbial community within the human intestine is incredibly complex and diverse, consisting of at least 10^14^ bacteria and archaea and approximately 1,100 prevalent species, with an average of 160 such species per individual [5, 6]. This microflora is estimated to possess 150 times more genes than human host genomes [7] and plays a crucial role in regulating human physiological and metabolic functions [8]. Recently, research on the modulation of the gut microbiota has focused on fecal microbiota transplantation (FMT), which can reverse intestinal microbiome dysbiosis in the recipient [9–12]. Given its advantages, FMT is considered an innovative and effective technique for the treatment of challenging diseases [13, 14], although there is controversy regarding FMT as a safe treatment technique [15].

Through animal studies, researchers have found that FMT can alter body phenotypes. Germ-free mice that received microbiota from human donors with obesity became obese, while those that received microbiota from human donors who had undergone bariatric surgery remained lean [16–18]. Studies have shown that FMT from lean donors can increase intestinal microbial diversity and improve insulin sensitivity in patients suffering from metabolic syndrome [19]. However, these studies have mainly focused on obese animals, and the transplantation of gut microbiota between naturally obesity-resistant and HFD animals has not been reported.

The house musk shrew, *Suncus murinus* (*S*. *murinus*), is an insectivorous animal that has been used in various fields of medicine due to its unique characteristics compared to laboratory rodents [20]. Our team has been mainly concerned with the peculiarities of its digestive system [21–25], and our research in recent years has shown that *S*. *murinus* is a naturally obesity-resistant experimental animal [26]; in particular, its resistance to mesenteric fat accumulation is a desirable trait not found in general animals, including humans, that stimulated our research interest. We previously found that even on an HFD, this animal was able to maintain its body weight and mediate fat gain by controlling its energy intake [27]. A further study found that the gut microbiota of *S*. *murinus* differs markedly from that of the general experimental animal C57BL, and the influence of antibiotic use for FMT on the transplantation effect was discussed [28].

In the present study, to further our research on naturally obesity-resistant animals, we focused on the microbes that inhabit the gut of *S*. *murinus*, we explored whether or not the ecological diversity of gut microbiota of obese mice fed an HFD could be improved by transplanting *S*. *murinus* gut bacteria.

## Materials and methods

### Animals

All animal experiments were approved by the Institutional Animal Care and Use Committee of Tokyo Metropolitan University, and all experiments were conducted in accordance with the National Research Council Guide for Care and Use of Laboratory Animals (A3-22, A4-19).

#### *S*. *murinus*

Male house musk shrews, *S*. *murinus* (n = 6, 4 weeks old), obtained from a closed breeding colony (JIc: KAT-c) at our laboratory were employed as donors for FMT experiments in this study. All animals were housed in polycarbonate cages in a room maintained at 28 ± 2°C with 50% ± 5% relative humidity in the Functional Morphology Laboratory, Department of Frontier Health Sciences, Tokyo Metropolitan University (Tokyo, Japan). *S*. *murinus* individuals were randomly swapped between cages three times per week for three weeks prior to the FMT procedure to minimize other variables that might cause differences in the microbiota. The room was automatically lit between 09:00 and 21:00. The pellets consisted of 45.0% protein, 4.0% fat, 3.0% fiber, 15.0% ash and 26.2% complex carbohydrates (Oriental Yeast Co., Ltd. Bioindustry Division, Chiba, Japan), and the metabolizable energy content was 357 kcal/100 g. Pellets and water were supplied *ad libitum* [28].

#### C57BL/6NCrSIc mouse

As recipients of FMT, specific-pathogen-free C57BL/6NCrSIc mice (male, n = 18, 4 weeks old) obtained from Sankyo Labo Service Corporation, Inc. (Tokyo, Japan) were housed in high-efficiency, particulate air-filtered cages with sterilized bedding with *ad libitum* access to food and water. All mice were kept on a 12-h light/dark cycle at 25 ± 2°C with humidity (50% ± 5%) in our laboratory. The HFD (60 kcal% fat, D12492; Research Diets Inc., New Brunswick, NJ, USA) was replaced every 2 days to keep it fresh and the HFD feeding period was from beginning to the end of the experimental. To avoid cross-infection of intestinal flora, recipient mice were kept in a private room after transplantation.

### Experimental design and procedure

**Fig 1:**
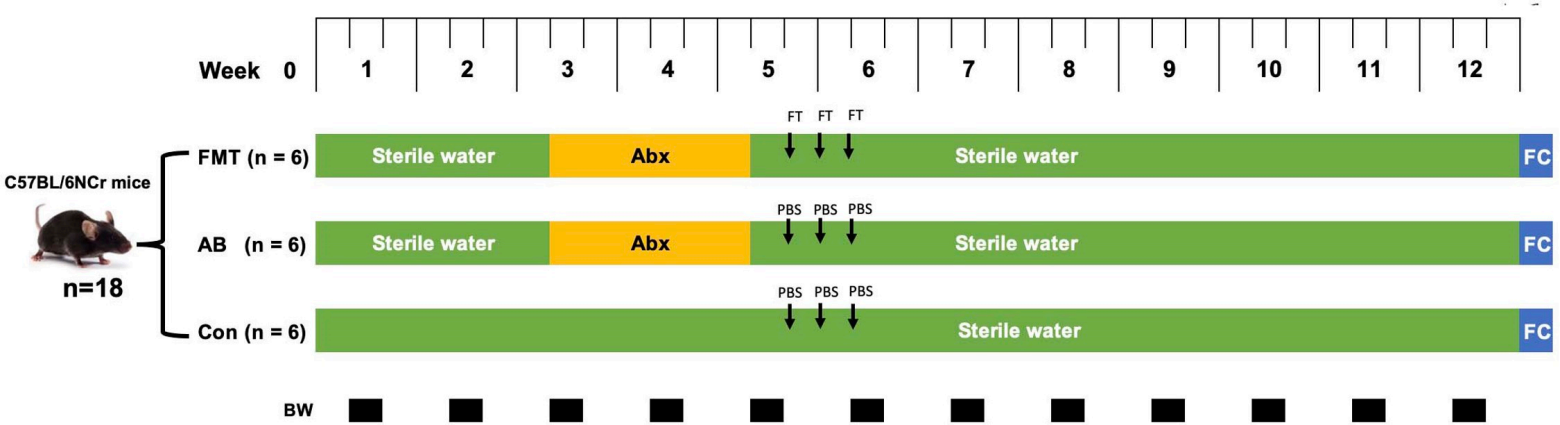
Schedual of experimental in each mouse group. Abx: antibiotic treatment period; FC: fecal microbiota collection; BW: body weight measurement; FT: fecal microbiotal transplantation from *S*. *murinus*; PBS: phosphate buffer saline. FMT: fecal microbiota transplantation group; AB: antibiotics group; Con: control group.

C57BL mice were randomly assigned to three groups: the control group (Con group, n = 6), the antibiotic administration group (AB group, n = 6) and the FMT group (FMT group, n = 6). All groups were housed in high-efficiency, particulate air-filtered cages with sterilized bedding with *ad libitum* access to food and water. The FMT group first received 10 days of antibiotic treatment, and after 3 days of drug withdrawal, the group received daily FMT from *S*. *murinus* for 3 consecutive days (Fig 1). The AB group received the same antibiotic treatment but no transplantation. The Con group received neither antibiotic treatment nor transplantation. Antibiotic cocktail including ampicillin (1.0 g/L; Nacalai Tesque, Kyoto, Japan), vancomycin (0.5 g/L; Shionogi, Osaka, Japan), neomycin (1.0 g/L; Nacalai Tesque, Kyoto, Japan) and metronidazole (1.0 g/L; Nacalai Tesque, Kyoto, Japan) in drinking water [28].

### FMT

The FMT procedure was conducted in line with our previous research [28]. In brief, to collect fecal content from the donor animal, all surgical instruments were aseptically sterilized before the transplantation surgery. Age- and sex-matched donor animals (*S*. *murinus*) were deeply anesthetized under inhalation anesthesia with isoflurane (concentration 5.0%, v/v) using a continuous inhalation anesthesia machine (SN-487-OT Air, WAKENYAKU CO., LTD, Kyoto, Japan), and then the abdominal skin was disinfected with iodine, and a midline abdominal incision was made. The large intestine (*S*. *murinus* has no cecum) was cut open, and approximately 100 mg of content was collected and transferred into a 5 ml sterile test tube, with care taken to avoid external contamination. One hundred milligrams of fecal content was collected and resuspended in 1 ml of sterile phosphate-buffered saline (PBS; pH = 7.2) (containing 10% triglycerides) in a 5 ml sterile microtube, mixed with a vortex mixer for 10 s and centrifuged at 805 × *g* for 3 min at 4°C to separate the supernatant that contained most of the microbiota from the solid fecal matter. Approximately 600 μl of the supernatant was removed and divided into 3 equal parts (200 μl/per tube), with 1 vial immediately available for transplant experiments and the other 2 stored at −80°C in a freezer for use over the next 2 days. Two hundred microliters of intestinal content supernatant was administered by oral gavage for three days. The FMT group continued to be fed sterile water and an HFD after transplantation until the end of the experiment. The mice in the Con and AB groups received oral gavage with an equal amount of PBS to match the stress of gavage manipulation during the same three-day period. After completing sample collection from the donor animals, the donor animals were euthanized by overdose anesthesia.

### Body weight

The body weight was measured twice a week (Fig 1).

### Extraction of DNA from feces

Eight weeks after FMT, three groups of mice were anesthetized until they reached a state of lethargy. Under as sterile a condition as possible, the abdominal cavity of the mice was opened, and the cecum was cut open. Approximately 200 mg of feces was collected from the cecum, and the collected feces were immediately dissolved with reagents from the DNA Kit. Fecal DNA was then extracted from the fecal samples using the ISOSPIN Fecal DNA Kit (Nippon Gene Co., Ltd., Toyama, Japan) according to the manufacturer’s protocols. The concentration of the extracted DNA was detected using a NanoDrop 2000 spectrophotometer (Thermo Fischer Scientific, Wilmington, DF, USA). Any samples that did not meet the detection standards were removed, and all DNA samples were immediately stored at -80°C before the next process.

### Library construction and sequencing

Paired-end (2×300 bp) sequencing was performed by Macrogen (Seoul, South Korea) using the MiSeq™ platform (Illumina, San Diego, CA, USA). The sequencing libraries were prepared using the Illumina 16S Metagenomic Sequencing Library protocols to amplify the V3–V4 region. The gDNA input of 2 ng was amplified by polymerase chain reaction (PCR) with Herculase II fusion DNA polymerase (Agilent Technologies, Santa Clara, CA, USA) and 5× reaction buffer, 1 mM dNTP mix and 500 nM each of the universal F/R PCR primers. The 1st PCR cycle conditions included 3 min at 95°C for heat activation, followed by 25 cycles of 30 sec at 95°C, 30 sec at 55°C, and 30 sec at 72°C, and a final extension of 5 min at 72°C. The universal primer pair with Illumina adapter overhang sequences used for the first amplifications was V3-F: 5’-TCGTCGGCAGCGTCAGATGTGTATAAGAGACAGCCTACGGGNGGCWGCAG-3’ and V4-R: 5’- GTCTCGTGGGCTCGGAGATGTGTATAAGAGACAGGACTACHVGGGTATCTAATCC-3’. The 1st PCR product was purified with AMPure beads (Agencourt Bioscience, Beverly, MA, USA). After purification, 2 μL of the 1st PCR product was PCR-amplified for the final library construction index using the NexteraXT Indexed Primer under the same PCR conditions as the 1st PCR, except that there were 10 cycles. The PCR product was purified with AMPure beads, and the final purified product was quantified by quantitative PCR (qPCR) according to the qPCR Quantification Protocol Guide (KAPA Library Quantification kits for Illumina Sequencing platforms) and qualified using TapeStation D1000 ScreenTape (Agilent Technologies, Waldbronn, Germany).

### Sequence analyses

For adapter trimming, adapter sequences were removed, and regions where two reads overlapped were error correction using fastp (v. 0.19.7) [29]. Subsequently, the paired-end sequences created by sequencing both directions of the library using the FLASH software program (v.1.2.11) resulting in the raw library and single long reads [30].

Operational taxonomic units (OTUs) were clustered with a 97% similarity cut-off and used CD-HIT-OTU (cd-hit-otu-illumina-0.0.1, http://weizhong-lab.ucsd.edu/cd-hit-otu) [31]. A BLAST+(v2.9.0) search (Query coverage > 85% and identity > 85%) was performed on each 16S rRNA gene sequence against the RDP version 11 database (RDP version 11 Update 4: May 26) to obtain the classification information.

### Bioinformatics and statistical analyses

QIIME (V.1.9.1) [32] and R Language (version 3.4.4, https://www.r-project. org/) were used for sequencing analysis of gut microbiota. The alpha diversity of microbial communities was determined using different indices (Chao1, Shannon, Inverse Simpson, observed richness) and calculated using QIIME (V1.9.1). The significance of differences in α-diversity index and relative abundance of different taxonomic groups was determined using the Kruskal‒Wallis test and Wilcoxon rank-sum test. Furthermore, bacterial community diversity was analyzed using rarefaction plots and boxplots and displayed using the R software program (version 3.4.4). Beta diversity was measured using Bray‒Curtis dissimilarity, unweighted UniFrac, weighted UniFrac and Jaccard distance measures. Tests for microbial community composition dissimilarity between pairs of groups were performed using nonparametric multiresponse permutation procedures (MRPPs), analysis of similarities (ANOSIM), and nonparametric permutational multivariate analysis of variance with the adonis function (Adonis) [33, 34] in R (version 3.4.4). A principal coordinates analysis (PCoA) based on the Bray‒Curtis distance matrix was performed in R (version 3.4.4), and a PCoA figure was created using the R package “ggplot2”. Visualization via nonmetric multidimensional scaling (NMDS) was used to evaluate the structural variations in microbial communities and displayed using R (version 3.4.4). Distance-based methods, such as the arithmetic average (un)weighted paired group method (UPGMA) [35], have been used to perform cluster analyses based on the similarities and dissimilarities of bacterial communities in samples. Heatmaps (heatmaps of differential species abundance clustering) were created using the “pheatmap” R package. Venn diagram created in the R package “Venn Diagram” were used to illustrate unique OTUs and those shared between samples [34]. Linear discriminant analysis (LDA) effect size (LEfSe) analysis [36] was used to detect statistically significant differences in species between study groups.

Two-sided *t-*tests were used for the statistical analyses using R (version 3.4.4), and the results are presented as the mean with the standard error of the mean (SEM). *p* values of < 0.05 were considered statistically significant.

## Results

As shown in Fig 1, this study completely completed the planned research plan. Fecal microbiota transplantation was completed for 3 consecutive days in the FMT group, and cross-infection was avoided between each group. No deaths or infectious complications from FMT were observed in this study.

### Body weight changes in each experimental group

To determine whether or not FMT causes changes in the body weight of recipients, body weight was measured throughout the experiment. Compared with the Con group, the body weights of the AB and FMT groups decreased significantly after antibiotic administration but began to recover approximately four days after antibiotic withdrawal (S1 Fig).

The body weights showed no marked difference throughout the experimental procedure; however, there was a tendency for the body weight of the FMT group to be lower than that of the Con and AB groups after the FMT procedure. In addition, the AB group showed a higher increase rate than the FMT and Con groups (S1 Fig).

### Overview of the sequencing data

In total, after filtering for chimeric and low-quality OTUs, 337663 high-quality reads corresponding to 431 OTUs were identified, and 81 genera were annotated. Sequencing metrics from 16S rRNA gene sequencing (V3-V4 regions), the averaged coverage and subsampling were sufficient to describe the gut bacterial community according to sequence-based rarefaction curves (S2 Fig) after quality filtering. As shown by respective rarefaction curves (phylogenetic diversity whole tree index, Chao1 index, and observed species), the rarefaction curves showed the number of species/OTUs under different sequence numbers, indicating significantly fewer gut microbiota species/OTUs in the FMT group than in the Con group (*p* < 0.01) but more than in the AB group (*p* < 0.01) (S2 Fig).

### Bacterial composition and relative abundance

**Fig 2:**
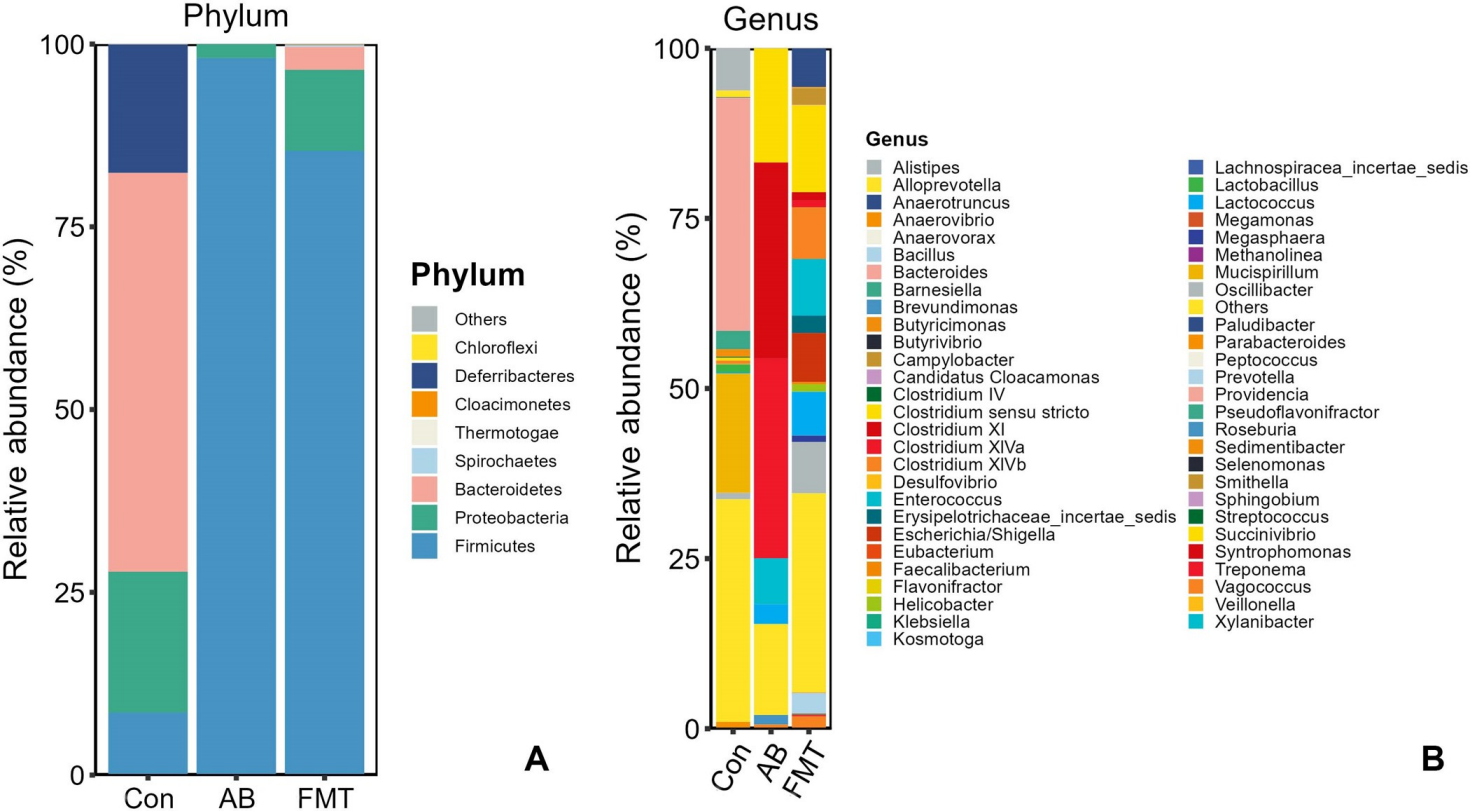
Relative abundance ratio of the intestinal microbiome at the phylum (A) and genus levels (B) in each experimental group. Data are presented as the percentage of species, and the results were obtained using the Kruskal–Wallis test. Con, control group; FMT, fecal microbiota transplantation group; AB, antibiotic group.

A total of 11 phyla, 20 classes, 26 orders, 46 families and 81 genera were detected in the prokaryotic microbiota communities from all fecal samples. We chose the main phyla and genera based on species abundance to generate a histogram, which showed the percentages of relative abundance in each sample or group (Fig 2).

At the phylum level (Fig 2A), the four most-abundant phyla were Bacteroidetes (54.73%), Proteobacteria (19.34%), Deferribacteres (17.47%) and Firmicutes (8.39%) in the Con group. In the FMT group, the most abundant phyla were Firmicutes (82.55%), Proteobacteria (11.00%) and Bacteroidetes (5.79%), and the abundance ratio of the remaining phyla was <1% in the FMT group, while the most abundant phyla in the AB group were Firmicutes (98.15%) and Proteobacteria (1.84%), with the remaining phyla accounting for <1%. The proportion of Firmicutes was significantly higher in the FMT group than in the Con group (*p* < 0.01), while there was no significant difference between the AB and FMT groups (*p* > 0.05). The proportion of Bacteroidetes in the Con group was significantly higher than in the FMT group (Fig 2A, *p* > 0.05), while Bacteroidetes was not present in the AB group at all. The Firmicutes/Bacteroides (F/B) ratio was significantly higher in the FMT group than in the Con group (Fig 1A, *p* < 0.05). In summary, the most common phyla in all groups (Con, FMT, and AB) were Firmicutes and Proteobacteria, while Deferribacteres was not detected in the FMT or AB groups, and Bacteroidetes was not detected in the AB group.

At the genus level (Fig 2B), the most abundant genera in the FMT group were *Prevotella* (5.38%), *Campylobacter* (2.9%), *Escherichia/Shigella* (6.68%), *Enterococcus* (8.01%), *Vagococcus* (1.68%), *Lactococcus* (5.89%), *Clostridium sensu stricto* (12.14%), *Clostridium XlVb* (7.29%), *Clostridium XI* (1.23%), *Anaerotruncus* (5.35%) and *Oscillibacter* (7.19%), most of which belonged to Bacteroidetes, Proteobacteria and Deferribacteres. In the AB group, the most abundant genera were *Enterococcus* (6.62%), *Lactococcus* (2.86%), *Clostridium sensu stricto* (17.21%), *Clostridium XlVa* (28.98%), *Roseburia* (1.43%) and *Clostridium XI* (28.94%), most of which belonged to Firmucutes. In the Con group, the most abundant genera were *Bacteroidales* (6.9%), *Barnesiella* (2.64%), *Alloprevotella* (1.05%), *Alistipes* (6.02%), *Bacteroides* (34.67%), *Mucispirillum* (17.47%), *Desulfovibrionaceae* (19.27%), *Lactobacillus* (1.08%) and *Lachnospiraceae* (2.55%), most of which belonged to Firmicutes and Proteobacteria.

**Fig 3:**
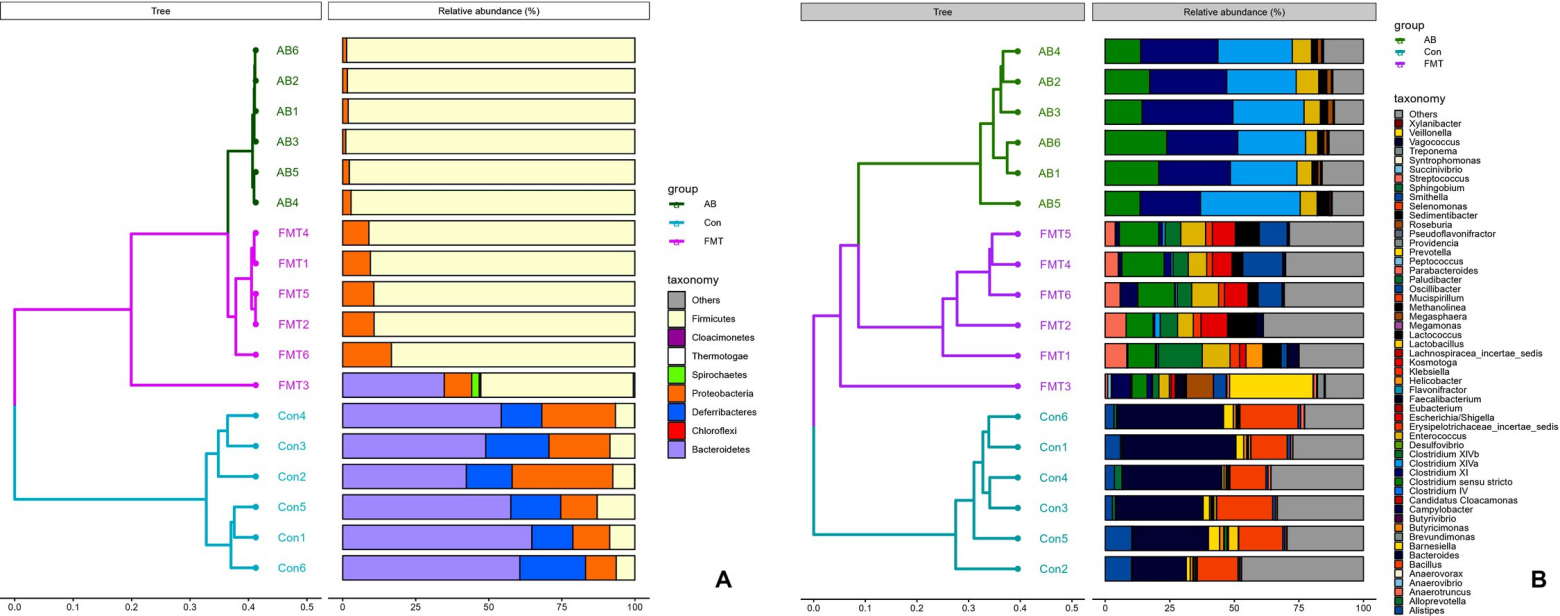
A cluster analysis based on the weighted Unifrac distance matrix in groups at the phylum (A) and genus levels (B). Control group: Con1, Con2, Con3, Con4, Con5 and Con6. Antibiotic group: AB1, AB2, AB3, AB4, AB5 and AB6. Fecal microbiota transplantation group: FMT1, FMT2, FMT3, FMT4, FMT5 and FMT6.

A hierarchical clustering analysis using the weighted Unifrac distance matrix was conducted following the UPGMA clustering method (Fig 3).

### Analyses of discrepancies between groups

**Fig 4:**
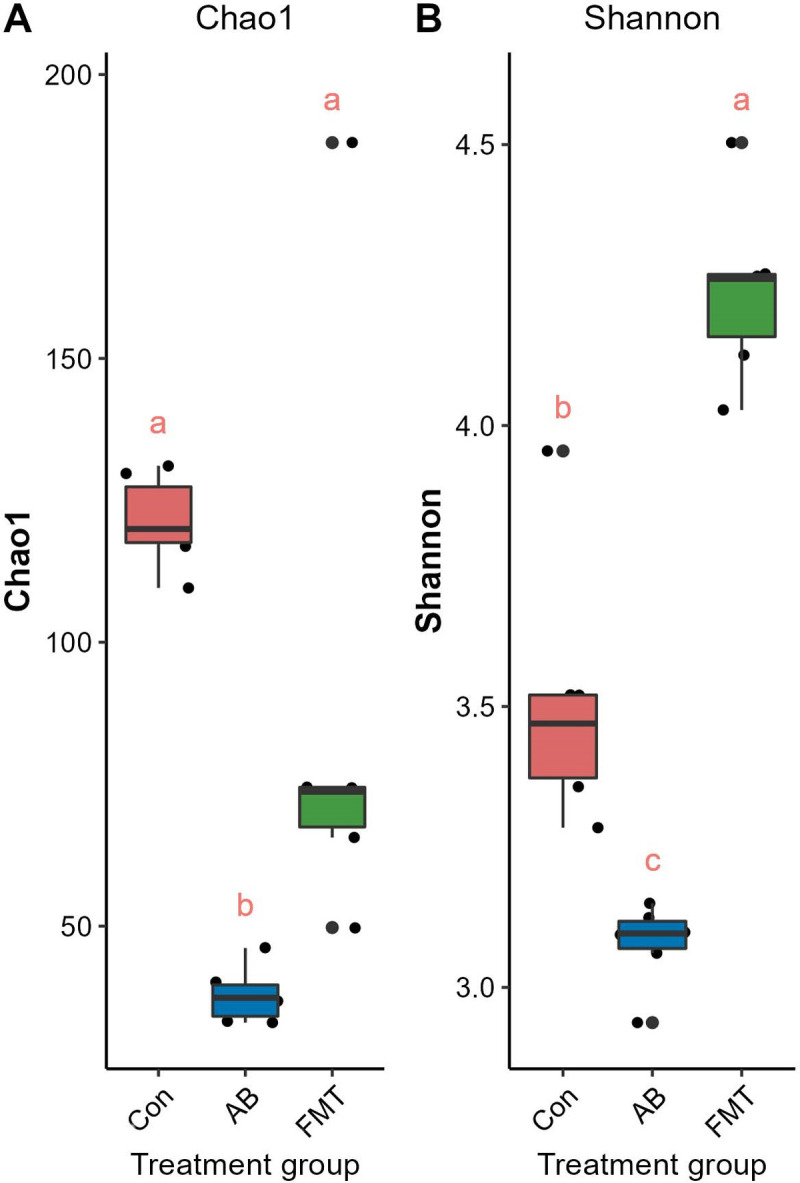
Comparisons of the alpha diversity, Chao1 (A) and Shannon (B) index. The same letter indicates that there was no significant difference between the groups; a different letter indicates a statistically significant difference between the groups. Con, control group; FMT, fecal microbiota transplantation group; AB, antibiotic group.

The comparison of the α diversity showed that there were significant differences in the species abundance (Chao1) and Shannon indices among the groups. The gut microbiota of the AB group featured a markedly decreased Shannon index and Chao index (Fig 4 and S1 Data) compared to the Con and FMT groups. The richness in the AB group was significantly lower than that in the Con and FMT groups, while there was no significant difference between the Con and FMT groups. A comparison of the diversity among those groups showed that the FMT group had significantly greater diversity than the Con and AB groups, and the AB group showed significantly lower diversity than the Con and FMT groups (Fig 4 and S1 Data).

**Fig 5:**
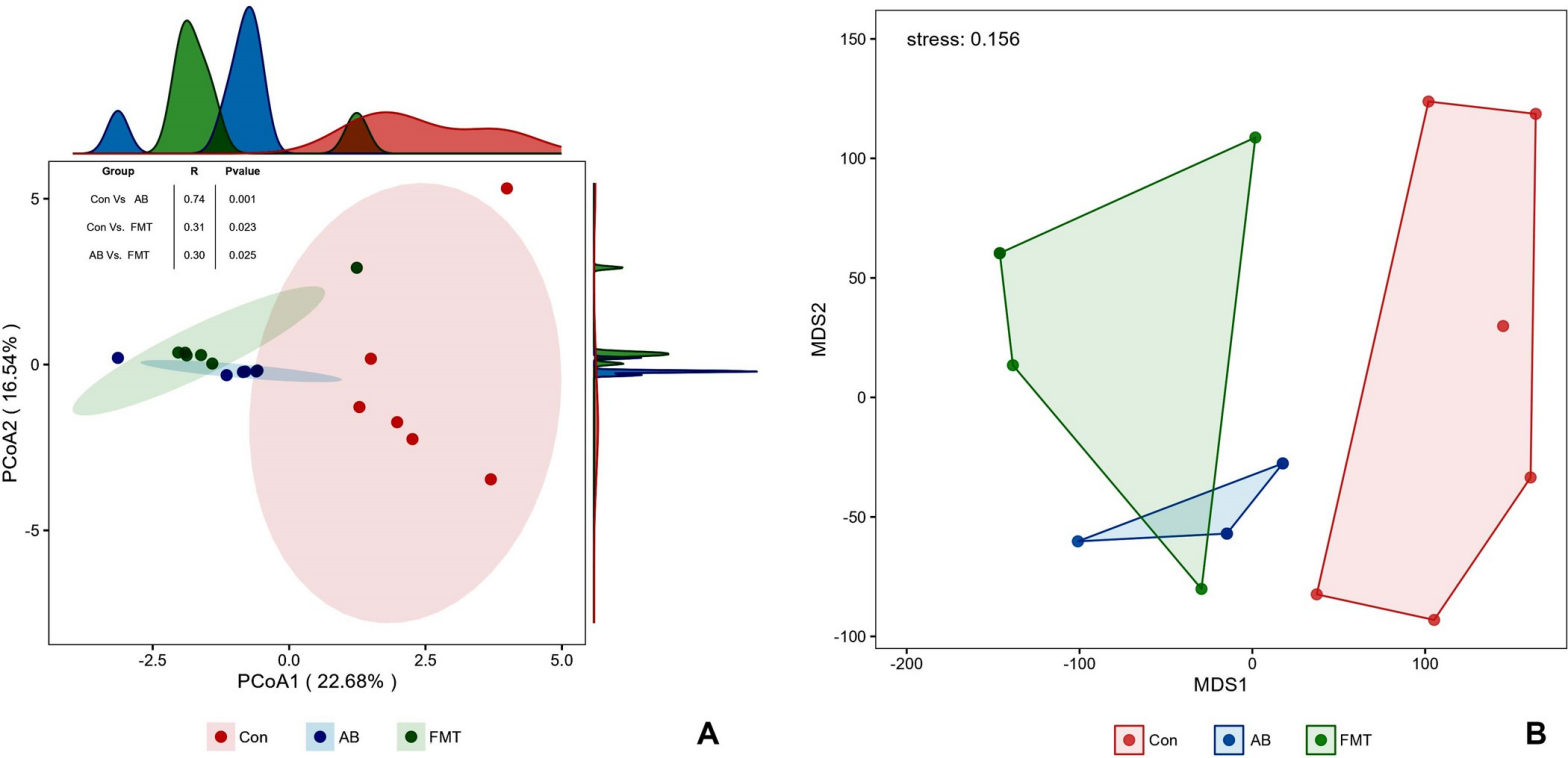
A: Principal coordinate analysis (PCoA) of the bacterial community structures of mouse gut microbiomes based on the Bray‒Curtis (weighted UniFrac) distance. (A-Box): The analysis of similarity (ANOSIM) of the three groups. B: Nonmetric multidimensional scaling (MDS) based on the abundance of OTUs. The closer the spatial distance of the sample, the more similar the species composition of the sample. Con, control group; FMT, fecal microbiota transplantation group; AB, antibiotic group.

Beta diversity analyses of the multiresponse permutation procedure (MRPP) (Table 1), based on the distance matrix, showed that vastly different bacterial community structures existed among the groups. A two-dimensional PCoA also revealed clear site-specific clustering with the different treatments (Fig 4A). In addition, An NMDS ordination plot (Fig 4B) of bacterial taxonomy data that demonstrated the microbial differences in samples and the distance between samples reflected the degree of the discrepancy. Combined with ANOSIM analysis (Fig 5A), the gut microbiota in the Con group proved significantly different from that in the AB group (*p* = 0.001, R = 0.74) or the FMT group (*p* = 0.023, R = 0.31), and the bacterial population structures in the samples from the AB and FMT groups (*p* = 0.025, R = 0.30) also showed statistically significant differences from Con group. This suggests that FMT or antibiotic treatment can change the microbiota composition in HFD-induced obese mice.

**Table 1:** The multiresponse permutation procedure difference analysis of the three groups.

Group	Distance	A	Observe delta	Expect delta	P value	P adj_BH
FMT vs. Con	Bray-Curtis	0.532050871	0.318192754	0.67997296	0.002	0.006
FMT vs. AB	Bray-Curtis	0.48982165	0.287108734	0.562761502	0.006	0.006
Con vs. AB	Bray-Curtis	0.690341792	0.19318747	0.623873241	0.005	0.006

Test of significance for the differences between groups using the multiresponse permutation procedures based on species abundance. Measure of distance based on Bray‒Curtis. A significance of delta (P_Value) of P < 0.05 indicates that the difference is significant. A = chance-corrected within-group agreement, A > 0 indicates that the difference between the groups is greater than the difference within the group, and A < 0 indicates that the intragroup difference is greater than the intergroup difference. Observe_delta, the smaller the observed delta value, the smaller the intragroup difference. Except_delta, the larger the value of except delta, the larger the difference between groups. Con, control group; FMT, fecal microbiota transplantation group; AB, antibiotic group.

### Bacterial composition difference analyses

**Fig 6:**
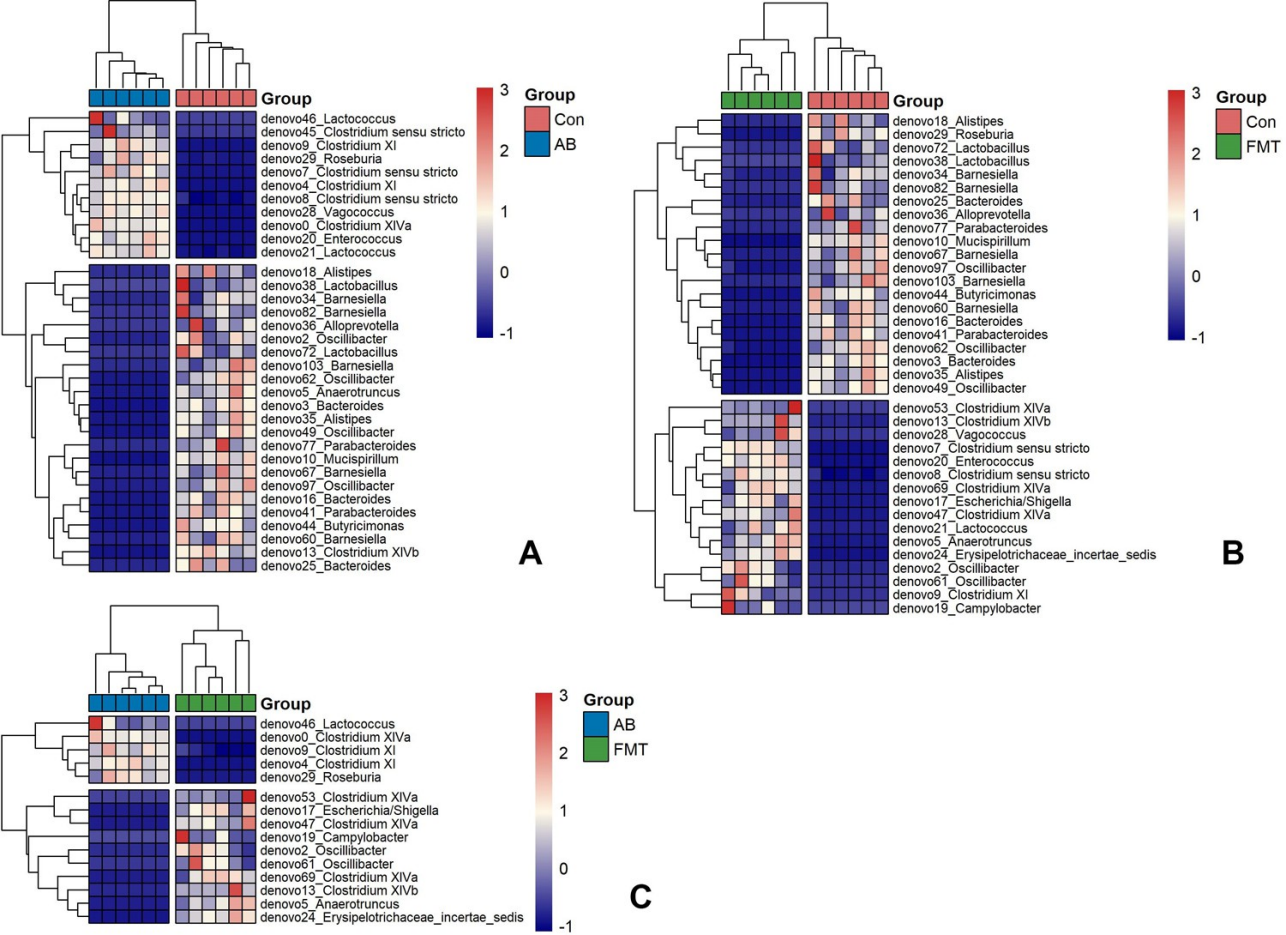
Heatmap of differential species abundance clustering between the AB and Con groups (A), the Con and FMT groups (B) and the AB and the FMT groups (C). The heatmap shows the hierarchical clustering of samples based on the relative abundance of fecal microbiota in the FMT, AB and Con groups. The relative values in the heatmap (after normalization), depicted by color, indicate the degree of aggregation or content of bacterial species among samples at the genus level. The color gradient from blue to red indicates low to high relative abundance, respectively. The vertical clustering indicates the similarity in the richness of different species among different samples. The closer the distance between two species, the shorter the branch length, indicating greater similarity in richness between the two species. Horizontal clustering indicates the similarity of species richness in different samples. Similarly, the closer the distance between two samples, the shorter the branch length, indicating greater similarity in species richness between the two samples. Con, control group; FMT, fecal microbiota transplantation group; AB, antibiotic group.

At the genus level, as shown in the heatmap (Fig 6) and LDA score (S3 Fig) between groups, there were 37 genera that differed significantly between the Con and FMT groups, of which 21 were significantly higher in the Con group, while 16 were significantly higher in the FMT group (Figs 6 and S3). In addition, 34 genera showed significant differences between the Con and AB groups, of which 23 were significantly higher in the Con group and 11 significantly higher in the AB group. Intriguingly, 15 genera showed significant differences between the AB and FMT groups, of which 10 were significantly higher in the FMT group and 5 were significantly higher in the AB group (Figs 6 and S3).

The common bacteria between groups were also analyzed using a Venn diagram (S4 Fig), which exhibited five common elements in "Con vs. AB", "Con vs. FMT" and "AB vs. FMT": *Denovo2_Oscillibacter*, *Denovo5_Anaerotruncus*, *Denovo9_Clostridium*, *Denovo13_Clostridium XIVb* and *Denovo29_Roseburia*. These analyses indicated that FMT from *S*. *murinus* influences the gut composition of mice.

We also performed an LEfSe analysis and identified bacterial genera that were significantly different among the groups (S3 Fig). These results were consistent with those of the heatmap analysis.

## Discussion

In the present study, FMT was conducted from the gut of *S*. *murinus* into the gut of C57BL mice fed an HFD to evaluate the effect of the procedure on improving the gut microbiota in these mice. 16S rRNA Illumina MiSeq high-throughput sequencing technology was used to perform a comprehensive analysis of the gut microbiota in C57BL mice, showing that the mouse group that received FMT from *S*. *murinus* had greater gut microbiota diversity than others.

In this study, there were no significant differences in body weight among the groups throughout the experiment; however, there was still a tendency for the body weight in the FMT group to be lower than in the Con and AB groups after the FMT procedure. This may be due to the influence of intestinal bacteria from the environment after FMT, which has a controlling effect on the weight gain of Recipients. It has also been reported that antibiotic use alone without transplantation resulted in weight gain relative to the transplantation group [37].

Analyses based on the operational taxonomic units (OTUs) showed significant changes in the gut microbial communities between mouse groups. Alpha diversity showed statistically significant differences among the FMT, AB and Con groups. The richness in the AB group was significantly lower than that in the Con and FMT groups, while there was no significant difference between the Con and FMT groups. Regarding the diversity among those groups, the FMT group showed significantly greater diversity than the Con and AB groups, while the AB group showed significantly poorer diversity than the Con and FMT groups. Furthermore, the beta diversity analysis indicated an apparent structural separation among the FMT, AB and Con groups, suggesting that FMT from *S*. *murinus* may affect the overall composition of the intestinal flora in C57BL mice.

In the present study, in terms of the alpha diversity, Chao1 and Shannon indices, the FMT group had greater gut flora diversity and richness than the AB group. A greater diversity is generally considered a mark of a healthy and resilient microbiota [38]. However, the Chao1 index revealed that the FMT group did not exhibit greater microbiota richness than the Con group, although it was higher than in the AB group. This was considered to be related to the dramatic decrease in richness after antibiotic treatment [39]. The antibiotic cocktail used in this study was active against a broad spectrum of Gram-negative, Gram-positive, and anaerobic bacteria and was regarded as suitable for clinical application. As expected, pretreatment with antibiotics decreased diversity and reduced the number of detected taxa. These findings are consistent with those of Dethlefsen and Relman [40], who reported a shift in the distal gut microbial composition and a decrease in diversity after antibiotic use. Interestingly, the microbiota diversity in the FMT group was significantly greater than that in the Con and AB groups, and the results indicated that FMT from *S*. *murinus* could help restructure the gut microbiota and improve the ecological diversity of the gut microbiota of the host.

Regarding the phylum diversity among the groups, the proportion of Firmicutes phyla (82.55%) in the FMT group was lower than that in the AB group (98.15%) but higher than that in the Con group (8.39%), while Deferribacters were not detected in the FMT or AB group. The AB group was deficient in Bacteroidetes, while Firmicutes was enriched in both the AB (98.15%) and FMT (82.55%) groups. It is postulated that a Firmicutes-dominant gut microbiota is associated with the development of obesity, while the relative abundance of Bacteroidetes increases as individuals lose weight [41]. The findings of a deficiency of Bacteroidetes in the AB group and lower proportion of Bacteroidetes (5.79%) in the FMT group than in the Con group (54.73%) are consistent with our previous study [28]. We speculate that Bacteroidetes might be very susceptible to antibiotics but could be reestablished by FMT.

The abundance of Proteobacteria was significantly lower in the AB group (1.84%) than in the FMT (8.21%) and Con (19.33%) groups (*p* < 0.01). Studies have shown that obesity is caused by the host’s resistance to leptin [42]. Elevated levels of leptin in host serum are accompanied by an increased abundance of Proteobacteria flora [43]. We thus inferred that FMT from *S*. *murinus* could help the FMT-treated mice re-establish their gut microbiota, which might help increase the abundance of Proteobacteria.

In addition, our results showed that the F/B ratio between groups was significantly different. Previous studies have shown that the F/B ratio is associated with obesity. However, a recent meta-analysis of mostly adult studies did not detect any such association [44]. In healthy mammals, the F/B ratio is relatively stable, and an increase or a decrease in the F/B ratio often implies a disease state [45]. Thus, the association between the F/B ratio and obesity remains controversial and may differ depending on the population studied. This study was consistent with a previous study, which found that antibiotic treatment resulted in an increased abundance of Firmicutes and a decreased abundance of Bacteroidetes [28]. Our study showed that after receiving transplants of fecal bacteria from *S*. *murinus*, the F/B ratio seemed to be reversed. We further found that the FMT group had a significantly higher F/B ratio (14.25) than the Con group (0.15). The F/B ratio in the FMT group was significantly lower than that in the AB group. Our findings suggest that FMT from *S*. *murinus* may have contributed to a rational improvement in the gut microbiota and healthy body condition in the FMT group.

The abundance of *Lactococcus* in the AB group was higher than that in the FMT group. This bacterium has been linked to conditions such as diabetes and obesity, and studies have shown that it primarily produces lactate during glucose metabolism. An increase in acetic acid-producing bacteria may contribute to obesity by enhancing the host’s ability to absorb energy from food [46, 47]. In contrast, the AB group had significantly higher levels of *Roseburia* than the Con group, while the FMT group had lower levels of *Roseburia* than the Con group. It was speculated that FMT from *S*. *murinus* might have suppressed the growth of *Roseburia*. *Anaerotruncus*, a genus of anaerobic bacteria, was significantly more abundant in the FMT group than in the Con and AB groups, possibly due to FMT from *S*. *murinus* promoting a healthier gut environment. *Anaerotruncus* may contribute to gut health and promote the growth of other beneficial bacteria.

*Campylobacter*, a Gram-negative bacterium, is involved in the production of short-chain fatty acids (SCFAs), such as butyrate, which are crucial for maintaining gut health and regulating the immune system [48]. In the present study, the FMT group had significantly higher levels of *Campylobacter* than the AB and Con groups, suggesting that accepting FMT from *S*. *murinus* may help improve the gut condition of mice. *Vagococcus* and *Enterococcus* are bacterial genera commonly found in the human gut and were shown to be enriched in the gut of *S*. *murinus* in our recent study [28]. As lactic acid-producing bacteria, they are believed to possess anti-obesity effects [37]. *Clostridium sensu stricto*, a group of anaerobic bacteria, is important for various functions in the gut, including the breakdown of complex carbohydrates and production of SCFAs. The FMT group showed significantly higher levels of *Vagococcus*, *Enterococcus* and *Clostridium sensu stricto* than the Con group, suggesting that FMT may have beneficial effects on the gut health and limit the side effects of antibiotics.

In conclusion, the innovation of this experiment was that we transplanted the intestinal flora of *S*. *murinus*, a naturally obesity-resistant experimental animal, into the intestines of HFD-induced obese mice. Although the body weight among the mouse groups did not change significantly, the HFD-induced obese mice that received gut microbiota from *S*. *murinus* showed a significantly improved gut composition and increased bacterial diversity, which can benefit the gut of mice. The mechanism underlying the relationship between the *S*. *murinus* gut flora and obesity resistance should be explored in a future study.

## Supporting information

S1 Fig The body weight curves of the FMT group, AB group, and Con group. Con, control group; FMT, fecal microbiota transplantation group; AB, antibiotic group. Abx start and Abx end indicate the start and end of antibiotic administration, respectively. Three arrows indicate the time point of the fecal microbiota transplantation.
(JPG)

S2 Fig The species rarefaction curve of each experimental group. This graph represents the numbers of observed species under different sequence numbers extracted randomly, Chao 1 (A), observed species (B) and PD whole tree (C). Chao1, Chao’s estimated richness; PD, phylogenetic distance; Con, control group; FMT, fecal microbiota transplantation group; AB, antibiotic group.
(JPG)

S3 Fig Taxa with significant differences found by a linear discriminant analysis (LDA) effect size (LEfSe) analysis. LEfSe results showing which bacteria were significantly different in abundance between groups. Histogram of log_10_(LDA scores) for features with differential abundance between the AB and FMT groups (A), the AB and Con groups (B) and the Con and FMT groups (C). Taxa of |log10(LDA scores)| >3 are presented. Con, control group; FMT, fecal microbiota transplantation group; AB, antibiotic group.
(JPG)

S4 Fig Venn diagram of shared and unique taxa at the genus level among the Con, AB and FMT groups. The numbers of shared and unique taxa in the three pairwise comparison groups are shown based on the operational taxonomic units. Con, control group; FMT, fecal microbiota transplantation group; AB, antibiotic group.
(JPG)

S1 Data Diversity and richness (mean ± SD) of the fecal bacteria communities of mice. OTUs, operational taxonomic unit; Chao1, returns the Chao1 richness estimate for an OTU definition; Shannon, the Shannon index takes into account the number and evenness of species; Inverse Simpson, the Inverse Simpson index represents the probability that two randomly selected individuals in the habitat will belong to the same species; Good’s Coverage, Coverage is calculated as *C = 1-(s/n)*. Con, control group; FMT, fecal microbiota transplantation group; AB, antibiotic group; SD, standard deviation.
(DOCX)
