# Supplementary figures and images for: Gut microbiota of Suncus murinus, a naturally obesity-resistant animal, improves the ecological diversity of the gut microbiota in high-fat-diet-induced obese mice
Source: PLoS One. 2023 Nov 22;18(11):e0293213. doi: 10.1371/journal.pone.0293213 (PMC10664932; doi:10.1371/journal.pone.0293213)

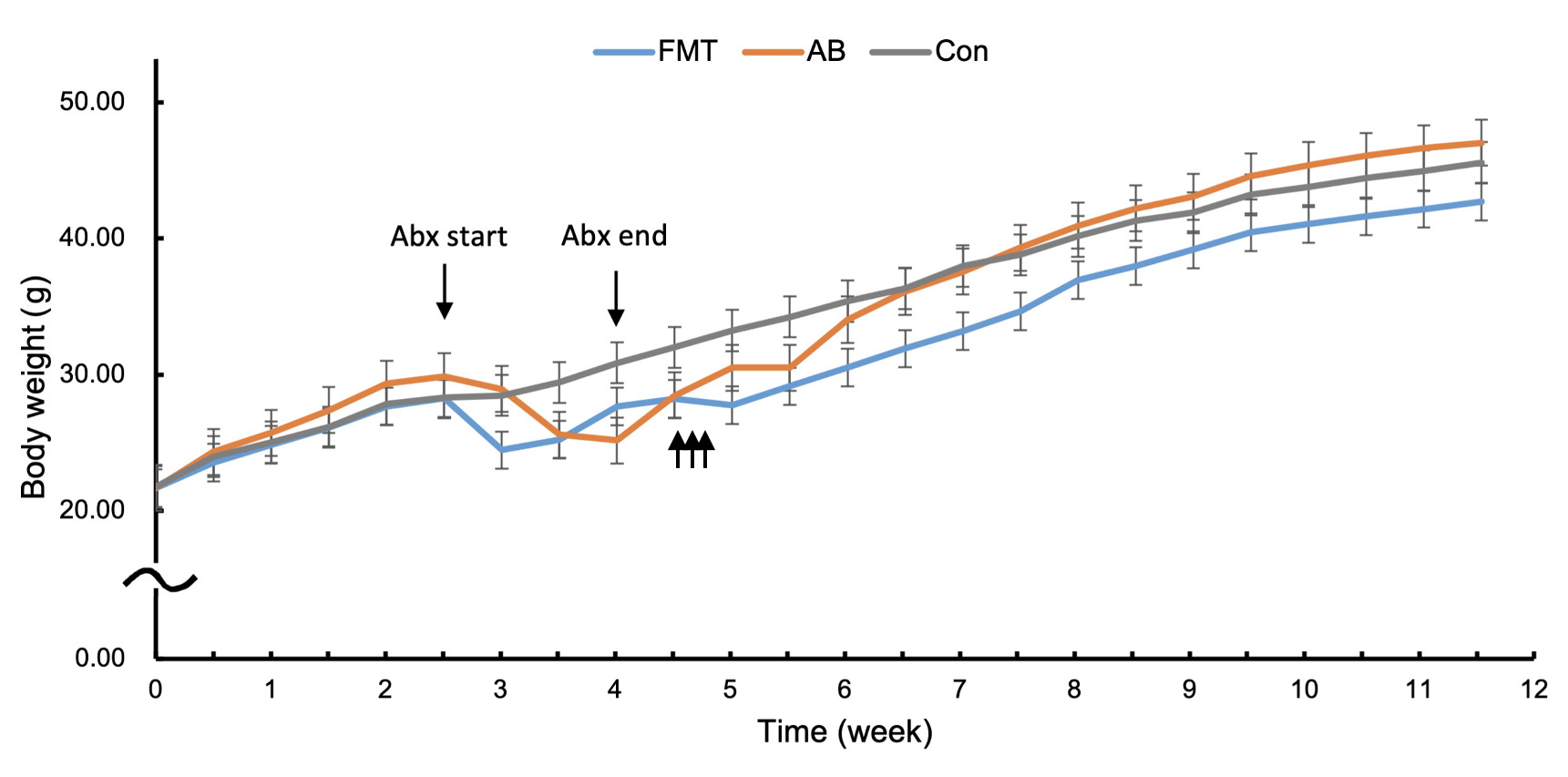

Supplement: S1 Fig — Con, control group; FMT, fecal microbiota transplantation group; AB, antibiotic group. Abx start and Abx end indicate the start and end of antibiotic administration, respectively. Three arrows indicate the time point of the fecal microbiota transplantation. (JPG) [file pone.0293213.s001.jpg]

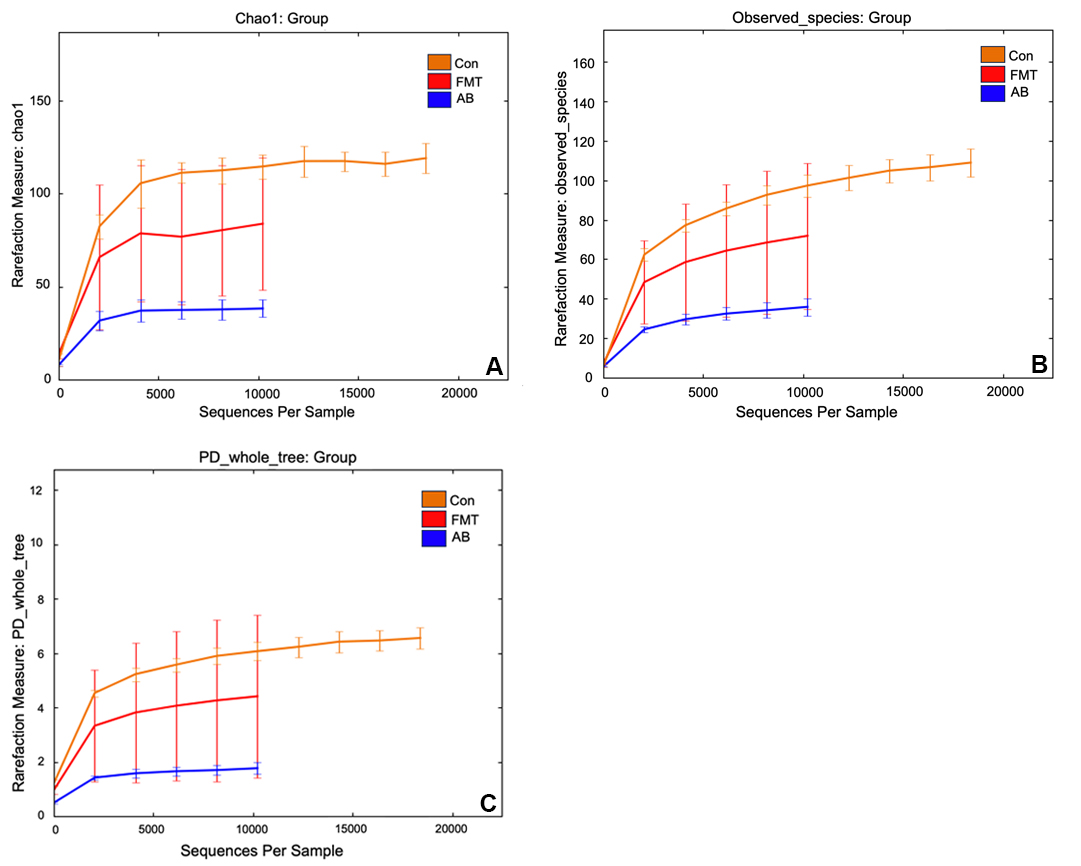

Supplement: S2 Fig — This graph represents the numbers of observed species under different sequence numbers extracted randomly, Chao 1 (A), observed species (B) and PD whole tree (C). Chao1, Chao’s estimated richness; PD, phylogenetic distance; Con, control group; FMT, fecal microbiota transplantation group; AB, antibiotic group. (JPG) [file pone.0293213.s002.jpg]

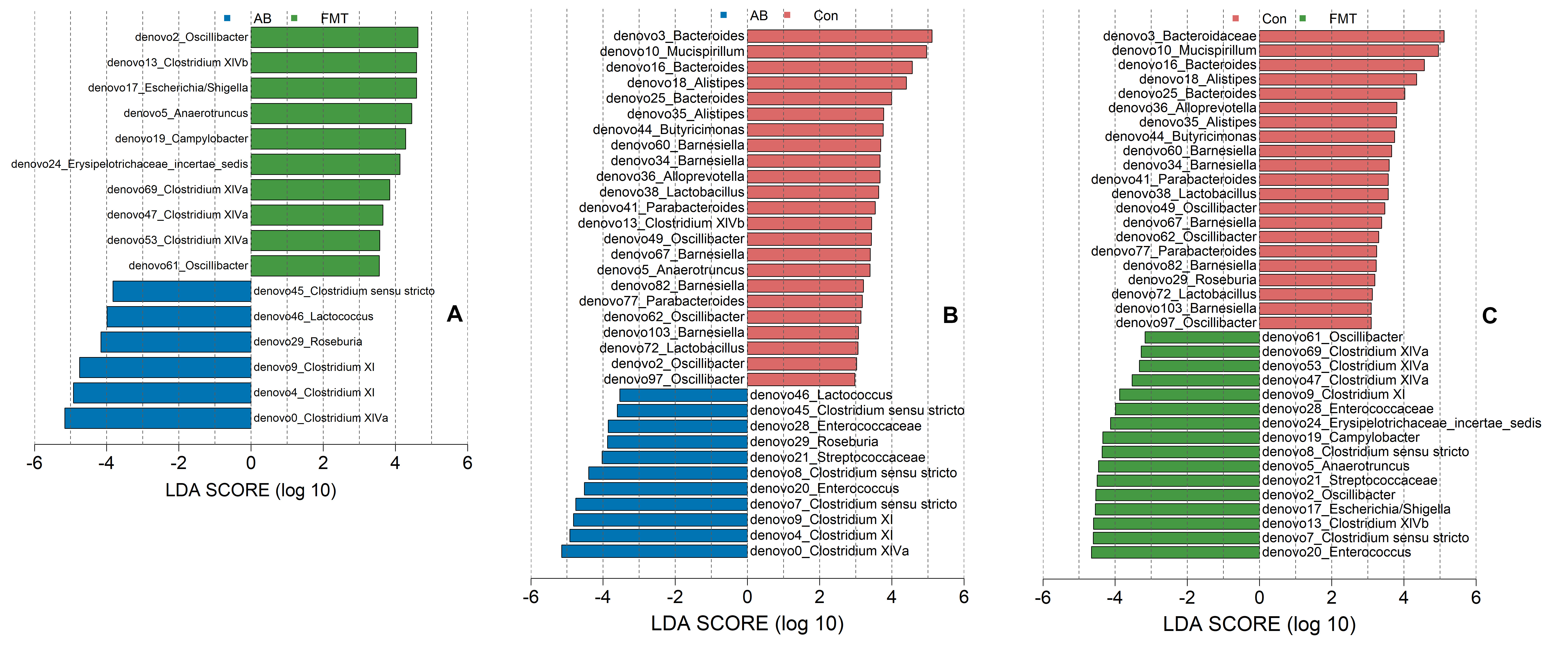

Supplement: S3 Fig — LEfSe results showing which bacteria were significantly different in abundance between groups. Histogram of log10(LDA scores) for features with differential abundance between the AB and FMT groups (A), the AB and Con groups (B) and the Con and FMT groups (C). Taxa of |log10(LDA scores)| >3 are presented. Con, control group; FMT, fecal microbiota transplantation group; AB, antibiotic group. (JPG) [file pone.0293213.s003.jpg]

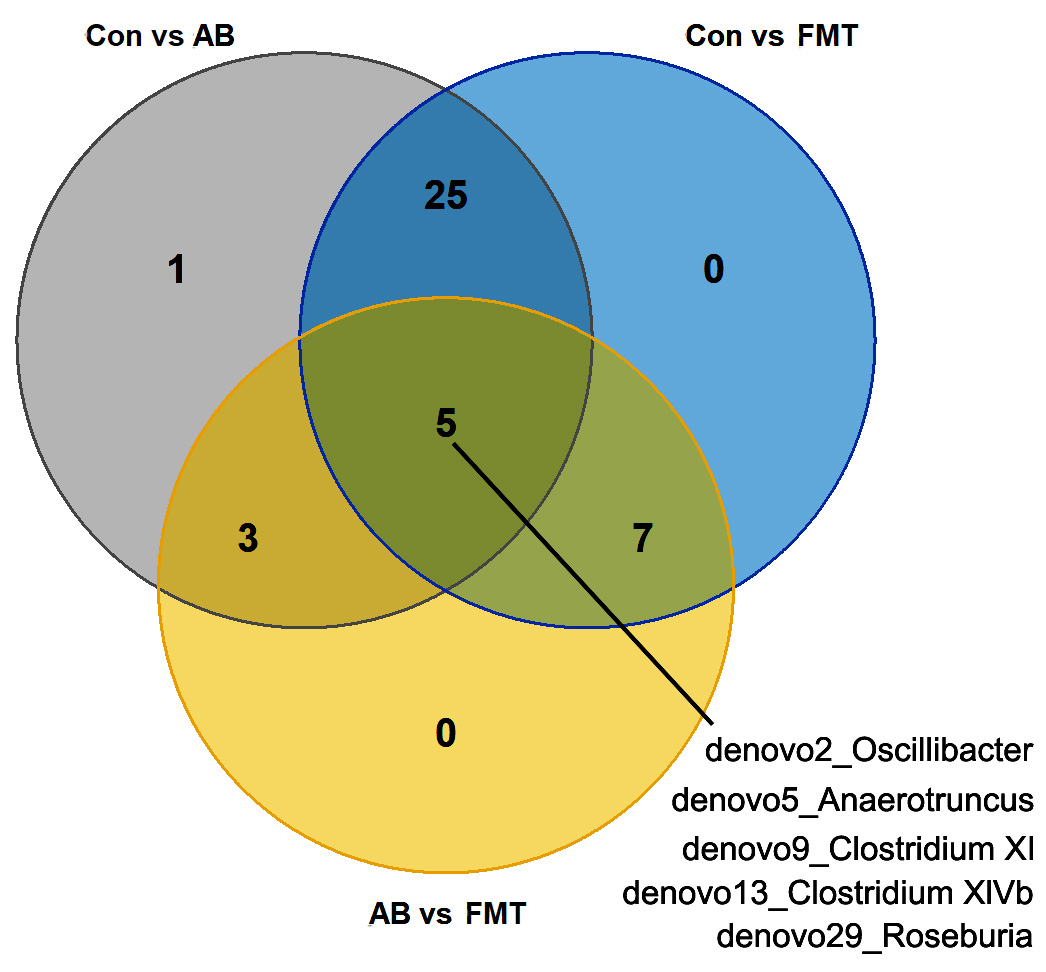

Supplement: S4 Fig — The numbers of shared and unique taxa in the three pairwise comparison groups are shown based on the operational taxonomic units. Con, control group; FMT, fecal microbiota transplantation group; AB, antibiotic group. (JPG) [file pone.0293213.s004.jpg]
