# Supplementary material for: Gut microbiota of Suncus murinus, a naturally obesity-resistant animal, improves the ecological diversity of the gut microbiota in high-fat-diet-induced obese mice
Source: PLoS One. 2023 Nov 22;18(11):e0293213. doi: 10.1371/journal.pone.0293213 (PMC10664932; doi:10.1371/journal.pone.0293213)
Supplement: S1 Data — OTUs, operational taxonomic unit; Chao1, returns the Chao1 richness estimate for an OTU definition; Shannon, the Shannon index takes into account the number and evenness of species; Inverse Simpson, the Inverse Simpson index represents the probability that two randomly selected individuals in the habitat will belong to the same species; Good’s Coverage, Coverage is calculated as C = 1-(s/n). Con, control group; FMT, fecal microbiota transplantation group; AB, antibiotic group; SD, standard deviation. (DOCX) [file pone.0293213.s005.docx]

**S1 Data.** Diversity and richness (mean ± SD) of the fecal bacteria communities of mice

|  |  |  |  |  |  |
| --- | --- | --- | --- | --- | --- |
| **Group** | **OTUs** | **Chao1** | **Shannon** | **Inverse Simpson** | **Good's Coverage** |
| **FMT** | 111.8 ± 7.94 | 121.19 ± 8.15 | 3.5093 ± 0.2384 | 0.8408 ± 0.0252 | 0.9992 ± 0.00 |
| **AB** | 37 ± 5.51 | 37.89 ± 5.49 | 3.0773 ± 0.0733 | 0.8387 ± 0.0192 | 0.9998 ± 0.00 |
| **Con** | 77.66 ± 38.33 | 87.53 ± 50.11 | 4.2414 ± 0.1609 | 0.924 ± 0.011 | 0.9994 ± 0.00 |

OTUs, operational taxonomic unit; Chao1, returns the Chao1 richness estimate for an OTU definition; Shannon, the Shannon index takes into account the number and evenness of species; Inverse Simpson, the Inverse Simpson index represents the probability that two randomly selected individuals in the habitat will belong to the same species; Good's Coverage, Coverage is calculated as *C=1-(s/n)*.

Con, control group; FMT, fecal microbiota transplantation group; AB, antibiotic group; SD, standard deviation.
